# Orthodontic Management of a Rare Incidence Bilateral Maxillary Canine-First Premolar Transposition Using Fixed Appliance

**DOI:** 10.1155/2022/9973333

**Published:** 2022-04-29

**Authors:** Samer Mereani, Amjad Alotaibi, Ahmed Bokhari

**Affiliations:** ^1^King Faisal Hospital, Makkah 24236, Saudi Arabia; ^2^Ministry of Health, P.O. Box: 43488, Al-Madinah 41311, Saudi Arabia; ^3^King Fahad Hospital, Jeddah 23325, Saudi Arabia

## Abstract

**Objective:**

A change in the location of two permanent teeth within the same quadrant of the dental arch is known as tooth transposition. This article illustrates the nonextraction treatment of a bilaterally complete transposition between maxillary canines and first premolars, using fixed mechanics. *Material and Method.* Upper and lower preadjusted, edgewise, fixed appliances with MBT prescription (0.022^″^ × 0.028^″^ slot size brackets) were used for the treatment. After providing room for the canine teeth, the piggyback technique was used to bring the right canine to the arch in the position of the first premolar. In the final phase of treatment, both the upper and lower arches had 0.019 × 0.025 stainless steel wire with Class II elastic 4 oz on the right and left sides.

**Result:**

It showed that the maxillary first premolars and canines were favorably aligned into transposed position. The upper right and left premolars occluded with the lower canines in Class I relationship and good intercuspation as well as skeletal and molar Class I relationships were maintained with a pleasant facial profile.

**Conclusion:**

The early diagnosis plays a significant role as much as considering esthetics and function factors in deciding which treatment strategy should be followed. The key to a successful and stable result is substantial treatment planning and careful orthodontic management.

## 1. Introduction

Tooth transposition is an uncommon dental anomaly and is usually related to the disturbance of eruptive position and tooth order [1, 2]. More precisely, Filho et al. described dental transposition as the interchange of the position between two permanents teeth, or the development and eruption of a tooth in a place ordinarily held by a nonneighboring tooth [3]. It is possible that the transposition is complete or incomplete. In the complete type, both the crown and root structures of the affected teeth are transposed. In the incomplete type, which is also known as simple, partial, coronal, or pseudotransposition, the crowns of adjacent teeth transpose, but the root apices remain in the normal tooth order [3]. According to most studies, the prevalence of transposition is less than 1% in the general population [4]. However, it varies according to the sample studied: a rate of 0.13% was reported in Saudi Arabia, 0.14% in Nigeria, 0.38% in Turkey, and 0.43% in an Indian population [5].

A recent study confirmed the dental transposition is a rare condition (0.66%) and presented no gender predilection [6]. Some authors did report a higher occurrence in females [4]. Furthermore, tooth transposition occurs in the maxilla more than in the mandible and more unilaterally than bilaterally, but no right or left side predisposition in the maxilla or mandible has been evident [4]. In contrast, many reports have found that the maxillary unilateral involved mainly the left side [7]. In bilateral cases, the symmetrical transposition is more observed than an asymmetrical transposition [4].

In the mandible, the most common transposition is between the canine and lateral incisor (Mn.C.l2), whereas in the maxilla, the most frequent transposition is between the canine and first premolar (Mx.C.P1) [5]. In spite of the (Max.C.P1) case being the most frequent form of tooth transposition, its incidence is very low in different populations. For instance, 0.03% of Swedish school children, 0.13% of Arabian dental patients, 0.25% of Scottish orthodontic patients, and 0.51% of individuals in a composite African sample [8].

Several theories have been suggested regarding the etiologic possibilities for dental transposition. Recently, a multifactorial etiology, both genetic and environmental factors, has been put forward as a theory to likely have been involved in the etiology of dental transposition [4]. Following a multifactorial inheritance model, there is a hypothesis that argues that Max.C.P1 transposition is genetically controlled, because it is frequently associated with other dental anomalies, familial occurrence, and the gender difference in its prevalence [4].

The decision on whether to keep or correct the transposed teeth is crucial to the treatment planning for transposition. From an orthodontic perspective, the treatment can be extraction or non-extraction. In the extraction option, either the transposed tooth is extracted, whereas in the nonextraction option, the transposed teeth are aligned in their normal positions or their transposed positions [3]. However, in most circumstances, especially in adult patients, there is a widespread agreement for accepting the transposed tooth order [7]. This case report demonstrates the management of a bilateral maxillary, permanent, and canine-first premolar transposition of a female patient.

## 2. Case Report

### 2.1. Diagnosis

**Figure 1:**
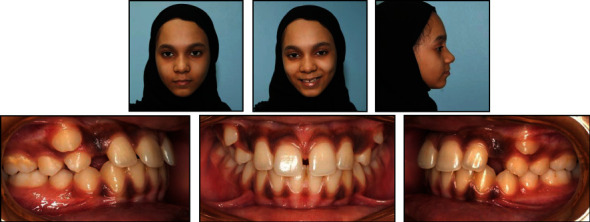
Pretreatment facial and intraoral photographs.

**Figure 2:**
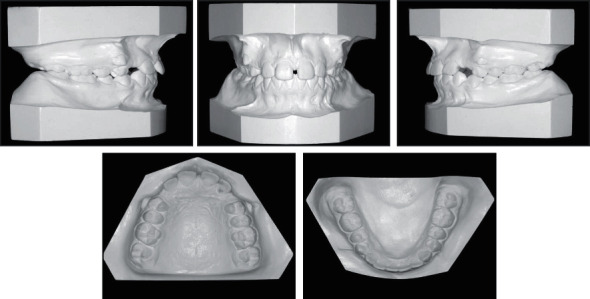
Pretreatment dental casts.

A female patient, aged 14 years and 3 months, reported to the department of orthodontics with a complaint of having an ugly smile because of the space between her front teeth. She had no previous orthodontic treatment or trauma history. Extraoral examination revealed a symmetrical and well-proportioned face pattern with a straight profile (Figure 1). The intraoral examination presented good oral hygiene with less attached gingiva at the upper canine area (Figure 1). Additionally, all permanent teeth, except the third molars, had erupted. The maxillary right and left permanent canines had partially erupted in an ectopic position at the buccal aspect in relation to the first premolars. She had a diastema (2 mm) with a high maxillary frenum, so the maxillary dental midline could not be determined. The interarch relationship was an Angle Class I molar relationship bilaterally, with 40% overbite and 4 mm overjet (Figure 2).

**Figure 3:**
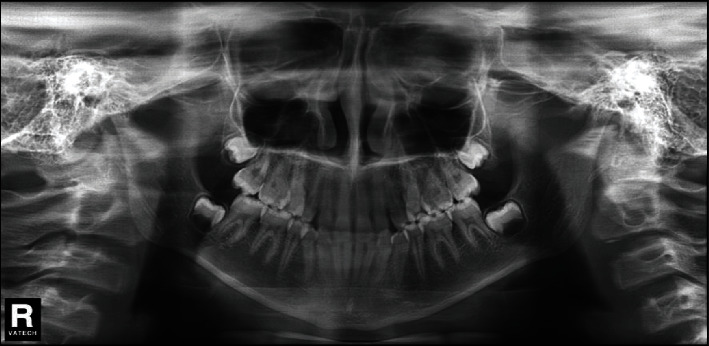
Pretreatment panoramic radiograph.

**Figure 4:**
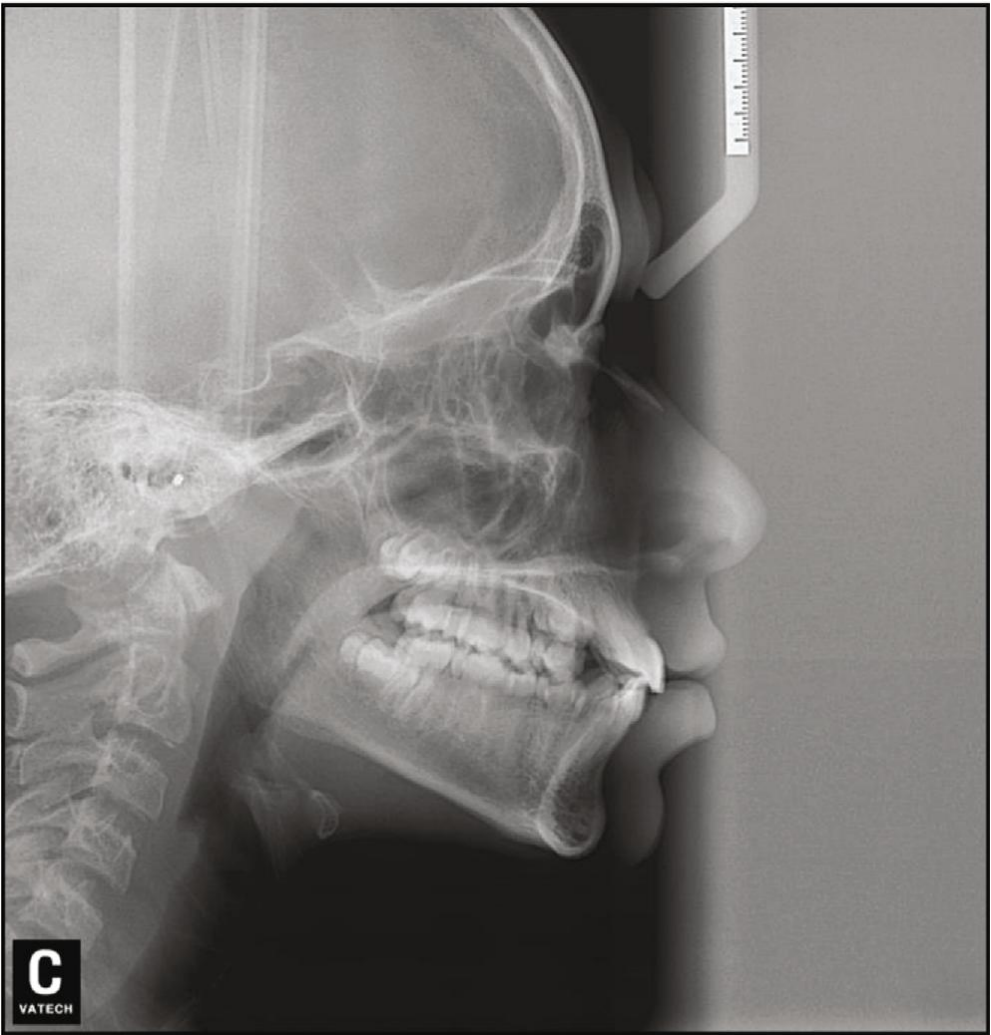
The lateral cephalometric radiograph.

**Figure 5:**
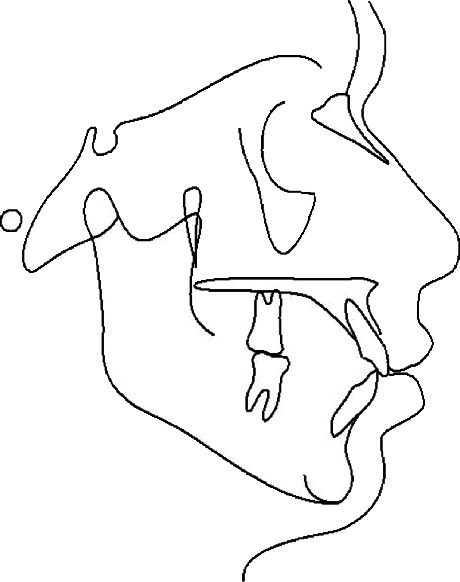
Initial cephalometric tracing.

The space analysis showed U-shaped form upper and lower arches, with mild crowding (1 mm) in the mandibular arch. On radiographic examination, there was a complete transposition of the crowns and the roots of the maxillary canine and first premolar (Max.C.P1) bilaterally, and with the roots of the canines were straight (Figure 3). The lateral cephalometric analysis revealed that, in this patient, there was a skeletal Class I relationship (*A* point, nasion, *B* point (ANB) angle = 2°) with normal upper and lower incisors (Figure 4). Normal upper and lower lips in relation to *E*-line were found with a normal nasolabial angle (Figure 5).

Several theories have been offered to explain the etiology of dental transposition. Recently, a multifactorial etiology, with both genetic and environmental factors, seems to be a likely theory in the etiology of dental transposition [5]. Following a multifactorial inheritance model, there is a hypothesis that argues Max.C.P1 transposition is genetically controlled, because it is frequently associated with other dental anomalies, familial occurrence, and the gender difference in its prevalence [3].

The patient was diagnosed with an Angle Class I malocclusion and bilateral transposition of the maxillary canine and first premolar, as well as mild crowding in the lower arch, based on earlier observations.

### 2.2. Treatment Objectives

The main goal was to achieve leveled and aligned teeth without compromising soft tissue and periodontal structure. For this reason, the treatment objectives were as follows: (1) to maintain the skeletal and molar Class I relationships, (2) accept Max.C.P1 transposition and establish premolars in the canine position into Class I relationship bilaterally, (3) correct the midline position in upper and lower arch to the facial midline, (4) resolve crowding in the lower arch, (5) maintain overjet and overbite within the normal range, and (6) to maintain the upper and lower lips' position in relation to the *E*-line and nasolabial angle.

### 2.3. Treatment Alternatives

The following alternatives can be carried out to solve this case: (1) the extraction of all upper and lower first premolars, (2) the nonextraction treatment and correction of the transposition, and (3) the nonextraction treatment and alignment of teeth in their transposed order.

### 2.4. Treatment Progress

After pretreatment records were taken and the analysis of diagnostic records was done, the importance of maintaining good oral hygiene and the benefits and risks of this treatment approach were presented to the patient and her guardians.

**Figure 6:**
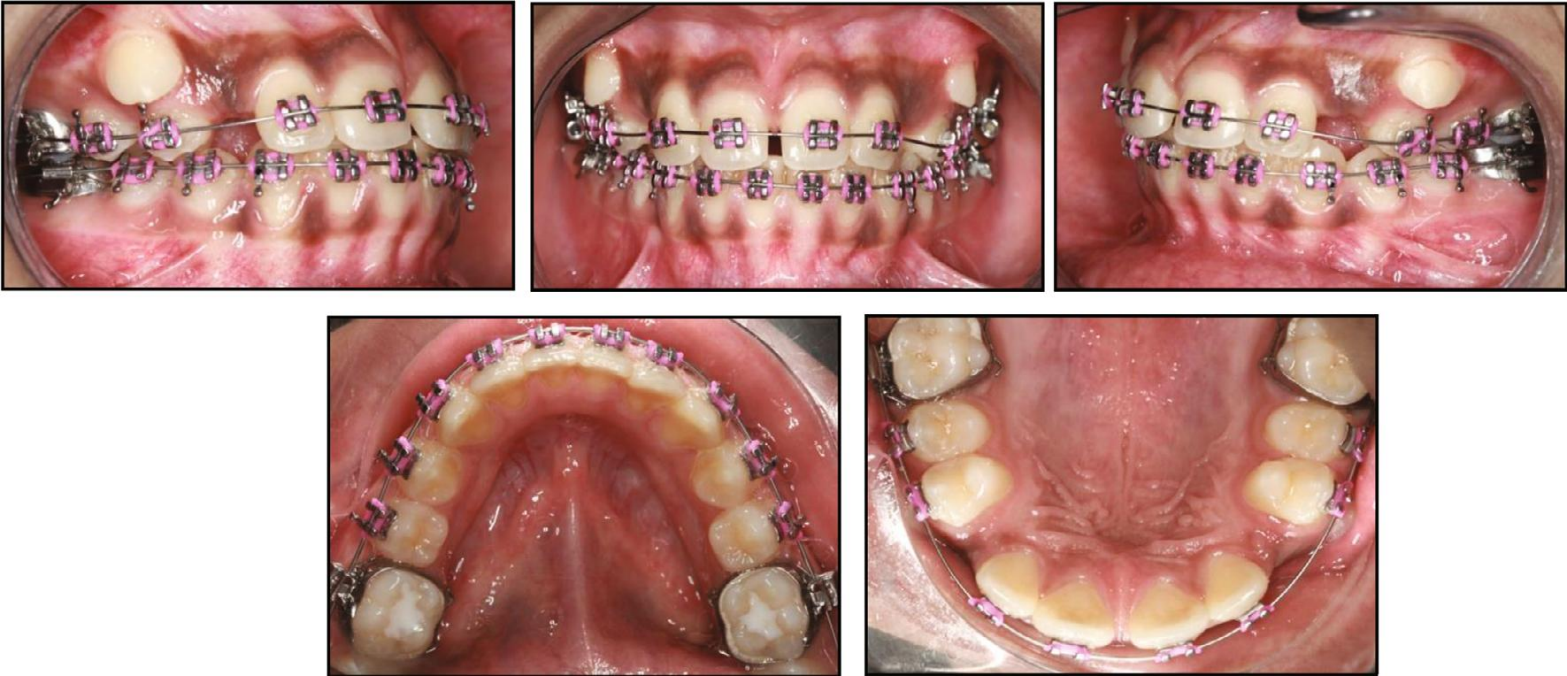
Banding and bonding (upper and lower preadjusted edgewise fixed appliances with MBT prescription and 0.022^″^ × 0.028^″^ slot size bracket) and placement of the initial leveling and aligning archwires (0.014 superelastic nickel titanium).

In the initial phases of treatment, the permanent molars were banded, and upper and lower preadjusted, edgewise, fixed appliances with MBT prescription (3 M gemini MBT 0.022^″^ × 0.028^″^ slot size brackets) were placed (Figure 6). During the bonding procedure, the first premolar crowns were bonded with the canine bracket to achieve the canine prominence (Figure 5). Alignment and leveling were accomplished using up to 0.019 × 0.025 nickel-titanium (3 M superelastic NiTi) in the upper and lower arches.

**Figure 7:**
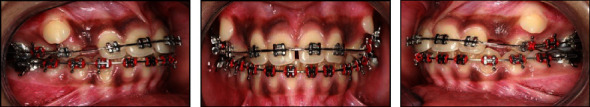
Nitinol coil spring between upper and second premolar.

**Figure 8:**
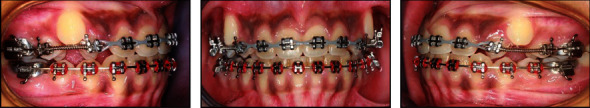
Powerchain from the upper right first premolar to left first premolar to close diastema.

In the maxillary arch, to provide room for the canine teeth, a 0.018 stainless steel archwire with a Nitinol coil spring was placed between the first and second premolars (Figure 7). Along with closing the diastema and moving the upper first premolars into the canine position, we used a power chain on braces from the right first premolar to the left first premolar (Figure 8). Steel colligation was used on the upper first and second premolars to avoid teeth rotation (Figure 8).

**Figure 9:**
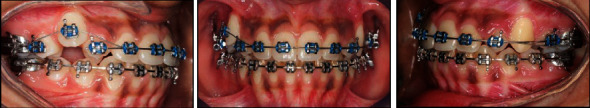
Piggyback 0.012 NiTi over 0.018 stainless steel archwire to bring the right canine to the arch.

**Figure 10:**
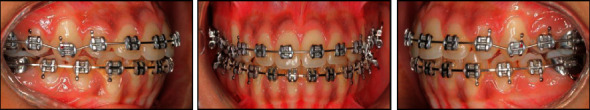
Both upper right and left canines included.

**Figure 11:**
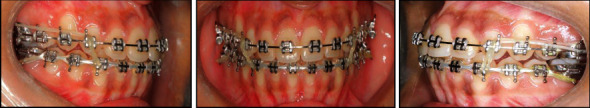
L-shaped elastic chain from lower canine to upper molar on both sides.

After 12 months, piggyback 0.012 NiTi over 0.018 stainless steel archwire was placed to bring the right canine to the arch in the position of first premolar (Figure 9). During this phase, the upper left canine was not included in the archwire and 0.019 × 0.025 stainless steel archwire used for the lower arch (Table 1). Both upper right and left canines were then included from 0.014 NiTi archwire up to 0.019 × 0.025 stainless steel archwire, with palatal root torque at the canines (Figure 10). Power chain was used from the mandibular right first molar to the left first molar, and an L-shape elastic chain (1/4 3 M medium force 4 oz) from the lower canine to the upper molar on both sides (Figure 11). The reduction of the palatal cusp of the upper right and left first premolars were also done. In the final phase of treatment, both the upper and lower arches had 0.019 × 0.025 stainless steel wire with Class II elastic 1/4 4 oz on the right and left sides.

**Figure 12:**
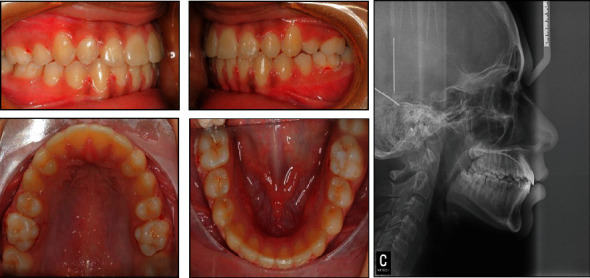
Posttreatment clinical photographs and lateral cephalogram.

**Figure 13:**
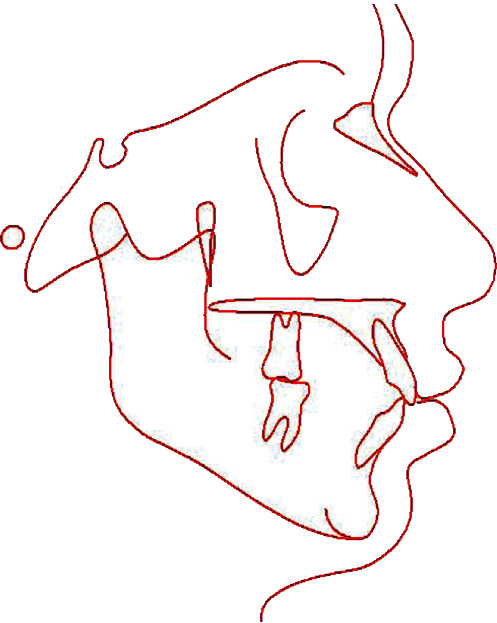
Final cephalometric tracing.

After 36 months of active treatment, the maxillary and mandibular fixed appliances were removed and the posttreatment records such as a panoramic radiograph (Orthophos XG), cephalometric X-rays (Orthophos XG), impressions, and photographs (Canon mark II with 90 mm macrolens) were taken to assess whether the treatment objectives were achieved (Figures 12 and 13). The patient was then instructed to wear upper and lower vacuum-formed retainers (Essex), and she was referred to restorative and periodontal clinics to improve her smile esthetics.

In considering these potential treatment options, the following factors were taken into account. Because the patient has a satisfactory facial appearance with a normal nasolabial angle and enough spaces at the upper arch, there was no need for teeth extraction. Moreover, keeping the position of the transposed maxillary canines was much easier than correction, as the canines would come down once enough spaces are created. In addition, her upper first premolar had a unique color and shape, which made it an ideal tooth for substituting the canine position. This may have reduced the difference between the canine and premolar, while making the final outcome insignificantly different than if the transposition had been corrected. The shorter treatment time with accepting the transposed tooth position was taken into consideration, because of the desire of the patient and her family. We, therefore, decided to attempt the nonextraction comprehensive orthodontic treatment, using upper and lower fixed appliances (preadjusted edgewise with McLaughlin, Bennett, and Trevisi (MBT) prospection and 0.022^″^ × 0.028^″^ slot size brackets).

## 3. Results

**Figure 14:**
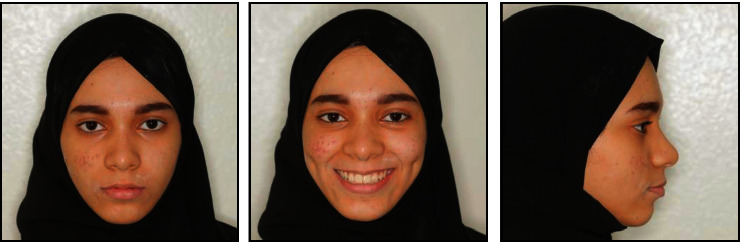
Posttreatment facial photographs.

**Figure 15:**
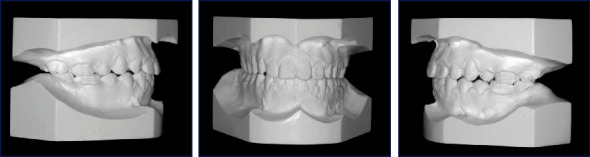
Posttreatment dental casts.

Skeletal and molar Class I relationships were maintained with a pleasant facial profile (Figure 14). The maxillary first premolars and canines were favorably aligned into transposed position. The upper right and left premolars occluded with the lower canines in Class I relationship and good intercuspation were obtained (Figure 15). All maxillary spaces, including the diastema, were closed and lower crowding was corrected. The upper and lower dental midlines were coincident with good overjet and overbite. From a periodontal point of view, all upper and lower teeth were well-positioned without supporting dental tissue complication, except for a mucogingival problem at the upper left and right canine areas, which was expected.

**Figure 16:**
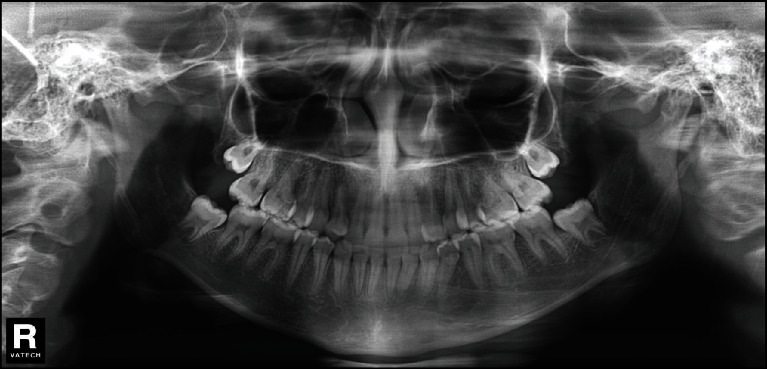
Posttreatment panoramic radiographs.

**Figure 17:**
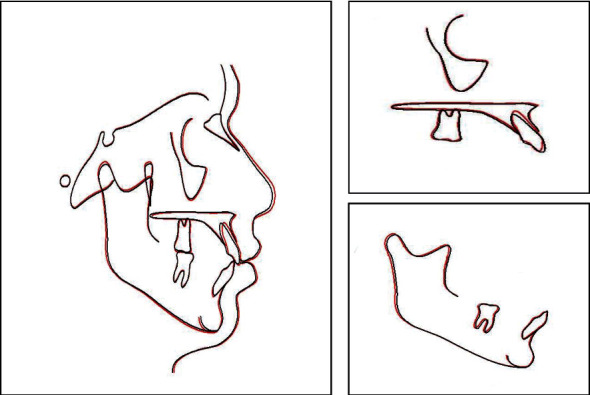
Pre- and posttreatment superimposition.

An end-treatment panoramic radiograph showed that all roots were in good paallelism, except for the upper left canine and the lower left first premolar (Figure 16). Cephalometric superimposition revealed that the upper incisors had retroclined and intruded about 2° (Figure 17). Furthermore, the mandible slightly had rotated downward and backward with the condylar head and the ramus showed some normal growth (Figure 17). The tip of the nose had grown forward and downward (2 mm). In general, the treatment was deemed successful and the treatment objectives were achieved. The patient was also fully satisfied with the results.

## 4. Discussion

The management of transposition should be performed on a case-by-case basis. Several therapeutic approaches to treating dental transposition were proposed in the literature. Extraction of one of the transposed teeth would reduce the time of treatment and make the correction of canine position easier. Other advantages were normal occlusal table contact and the elimination of needed esthetic and periodontal procedures in the future. Considering the facial profile, this approach would impair the patient's facial profile, which was already pleasant and straight. In the nonextraction strategy, there were two alternatives to manage this case, either maintaining the transposed order or correcting the transposition. Although correcting the order of transposition was the ideal treatment from functional and esthetic perspectives, this option has many disadvantages. For instance, treatment time was longer and the damage of supporting tissue and root resorption were possible; the mechanics were also complex and patient compliance was needed. On the other hand, keeping the teeth in their transposed position would require less time and simpler mechanics. This alternative poses some challenges regarding functional and esthetic outcomes.

In this case report, we decided to maintain the transposition, mainly because of the desire of the patient and her family to provide a definitive solution within less treatment time. Despite it being a simpler method of treatment, it was important to control the angulation and torque of the root of the first premolars and canines. With this in mind, the first premolar brackets were bonded to the canines to control their root torque and move them palatially to hide root bulge. The canine brackets were bonded to the first premolars to mimic the canine prominence and avoid functional interference. As the palatal cusp of the transposed premolar might have caused occlusal interference, it was reshaped to obtain optimal intercuspation. The similarity in shape, size, and tooth color between the first premolar and canine enhanced the smile esthetics. In the future, an optional prosthetic restoration and a gingival contouring procedure would ensure an optimal esthetic result. Nevertheless, the patient was pleased with her smile appearance and opted not to proceed with restorative and periodontal procedures. Satisfactory functional and esthetic outcomes were achieved without major injuries. These outcomes will give us some insight into the treatment options available for this dental anomaly.

Our therapeutic approach has certain limitations, such as requiring the patient to commit to a longer treatment duration (3 years), but it would guarantee a symmetrical outcome without the need for future restorative procedures. For instance, Palma et al. reported a 35-year follow-up of a very interesting case of bilateral Max.C.P1, associated with bilateral agenesis of the maxillary lateral incisors that were treated by maintaining the transposition [7]. Their result was both functionally and esthetically stable over time [7]. Another drawback in our case report is the loss of follow-up for such a long time, as the fact that follow-up visits are critical in determining a study's validity. In addition to this, orthodontic treatment can temporarily alter some variables such as teeth mobility due to long loading durations, and teeth sensitivity to thermal stimuli, especially in the anterior teeth, because of orthodontic debonding [9, 10]. Further long-term studies monitoring patients with transposition over time are needed in future clinical trials.

## 5. Conclusion

Dental transposition is a very rare and challenging situation in terms of treatment planning and management. When deciding which treatment strategy should be followed, the early diagnosis plays a significant role, along with considering esthetics and function factors. In general, the key to a successful and stable result is substantial treatment planning and careful orthodontic management.

## Figures and Tables

**Table 1:** The arch sequence for both arches.

Upper arch	Lower arch
0.014 superelastic NiTi	0.014 superelastic NiTi
0.016 NiTi	0.016 NiTi
0.018 NiTi	0.018 NiTi
0.018 SS	0.017 × 0.025 NiTi
Piggyback 0.012 NiTi over 0.018 SS	0.019 × 0.025 NiTi
0.014 NiTi	
0.018 NiTi	0.017 × 0.025 SS
0.017 × 0.025 NiTi	
0.019 × 0.025 NiTi	0.019 × 0.025 SS
0.019 × 0.025 SS	

NiTi: nickel titanium; SS: stainless steel.

## Abbreviations

Max.C.P1: The maxillary canine and first premolar
Mn.C.l2: The mandibular canine and lateral incisor.
